# Role of Gut Microbiome in Atherosclerosis: Molecular and Therapeutic Aspects

**DOI:** 10.2174/1573403X19666230202164524

**Published:** 2023-07-05

**Authors:** Juan Salazar, Valery Morillo, María K Suárez, Ana Castro, Paola Ramírez, Milagros Rojas, Roberto Añez, Luis D’Marco, Maricarmen Chacín-González, Valmore Bermudez

**Affiliations:** 1 Endocrine and Metabolic Disease Research Center, School of Medicine, University of Zulia, Maracaibo, Venezuela;; 2 Departamento de Endocrinología y Nutrición. Hospital General Universitario Gregorio Marañón, Madrid, España;; 3 Universidad Cardenal Herrera-CEU, CEU Universities, Valencia, 46115, Spain;; 4 Universidad Simón Bolívar, Facultad de Ciencias de la Salud, Barranquilla, Colombia

**Keywords:** Atherosclerosis, gut, microbiome, dysbiosis, inflammation, treatment

## Abstract

Atherosclerosis is one of the most relevant and prevalent cardiovascular diseases of our time. It is one of the pathological entities that increases the morbidity and mortality index in the adult population. Pathophysiological connections have been observed between atherosclerosis and the gut microbiome (GM), represented by a group of microorganisms that are present in the gut. These microorganisms are vital for metabolic homeostasis in humans. Recently, direct and indirect mechanisms through which GM can affect the development of atherosclerosis have been studied. This has led to research into the possible modulation of GM and metabolites as a new target in the prevention and treatment of atherosclerosis. The goal of this review is to analyze the physiopathological mechanisms linking GM and atherosclerosis that have been described so far. We also aim to summarize the recent studies that propose GM as a potential target in atherosclerosis management.

## 1 INTRODUCTION

Atherosclerosis is a chronic multifactorial inflammatory disease. Its pathological mechanism is mainly characterized by the initial formation of fat streaks in the tunica intima of arteries. This then progresses to atherosclerotic plaques formed by lipids, inflammatory cells, collagen, and cellular detritus. This triggers an inflammatory reaction that leads to endothelial injury, scarring, and thickening and hardening of the vascular walls. Depending on the size and stability of the plaques, these can completely or partially block arteries or they can detach and cause coronary events. These are the most common and lethal complications of atherosclerosis, and it has been estimated that these events could be the cause of approximately 12 million deaths by 2030 [1].

Atherosclerosis is currently considered the main precursor of cardiovascular disease (CVD), including coronary cardiopathy, cerebrovascular disease, and peripheral arterial disease. These constitute a significant economic burden for healthcare systems and are a global public health problem [2]. Due to its epidemiologic importance, research efforts have focused on detailing the subjacent molecular mechanisms in search of new therapeutic targets. Among these, the gut microbiome (GM) is considered a new factor to consider in the pathophysiology of atherosclerosis [3-5].

There has been substantial interest in the interaction between GM and the progression of atherosclerosis. Multiple studies have pointed out that changes in GM can affect cardiovascular outcomes [6]. Likewise, the presence of bacterial DNA corresponding to GM has been identified on atherosclerotic plaques. It has been demonstrated that GM can, directly and indirectly, affect the formation of these plaques through physiopathologic mechanisms that are currently under study [7]. These findings have led to the consideration of GM as a new therapeutic target for the management of atherosclerosis, specifically regarding its proinflammatory role, which is the key in this process [8].

Therefore, the goal of this review is to describe the molecular mechanisms that link GM with the pathophysiology and progression of atherosclerosis, proposing a potential therapeutic role in underlying inflammatory phenomena.

### 1.1 Microbiota and Atherosclerosis: Physiopathological Mechanisms

The GM plays an important role in body homeostasis. Some of its functions include the processing and fermenting of undigestable substances, the synthesis of certain metabolites and vitamins, and the production of short-chain fatty acids (SCFA). This allows the GM to participate in different metabolic routes as well as the regulation of the gut barrier [9, 10]. Therefore, any alteration of the GM could generate a significant impact on different functions of the body, breaching the epithelial barrier and allowing bacterial translocation with the subsequent absorption of their products [11]. This would lead to an inflammatory immune response and alterations in different metabolic pathways.

Considering that atherosclerosis is a disease characterized by chronic inflammation, it is logical to think that there could be a connection between this entity and gut dysbiosis (GD), a theory that has been supported by different studies [6, 7]. This has led to the proposal of different research lines to comprehend the GM and the pathophysiological phenomena of atherosclerosis. Possible direct and indirect mechanisms involving the production of microbe metabolites and local and systematic response, respectively, have been described.

#### 1.1.1 Infection-induced Inflammatory Response - Indirect Mechanism

Different studies have confirmed the presence of bacterial DNA in atherosclerotic plaques, with the Firmicutes and Proteobacteria phylum being the most abundant [12]. Many of these bacteria are part of the oral cavity and the gut, which are ideal places for the reservoirs of these microorganisms [13]. The processes through which the presence of infections can affect the development of atherosclerotic plaques are not entirely understood; however, two mechanisms have been proposed. The first one is the direct infection of the cells of the vascular wall, which creates an area vulnerable to the development of the lesion. The second one is the indirect infection that is found in a place distant from the atherosclerotic plaque, which is capable of activating the immune system, triggering a systemic inflammatory response [7].

The maintenance of the integrity of the gut barrier is one of the functions of the GM, which modulates the permeability of the barrier as well as the absorption of nutrients. This involves the drainage of lipids absorbed by the small intestine through the capillary and lacteal networks present in the gut villi [14]. Different studies have reported that diets rich in saturated fat can alter the normal GM by provoking a decrease in the number of Gram-positive bacteria and an increase in the proliferation of Gram-negative bacteria. The latter have lipopolysaccharides (LPS) as an essential component of their plasmatic membrane. These are the main pathogen-associated molecular patterns (PAMPs) derived from the GM [15]. Depending on the integrity of the gut barrier, these PAMPs can enter the blood circulation, triggering inflammatory signals in peripheral organs. This leads to chronic inflammatory processes, including inflammation of the white adipose tissue, brain cavernous malformations, and atherosclerosis [16].

**Fig. (1):**
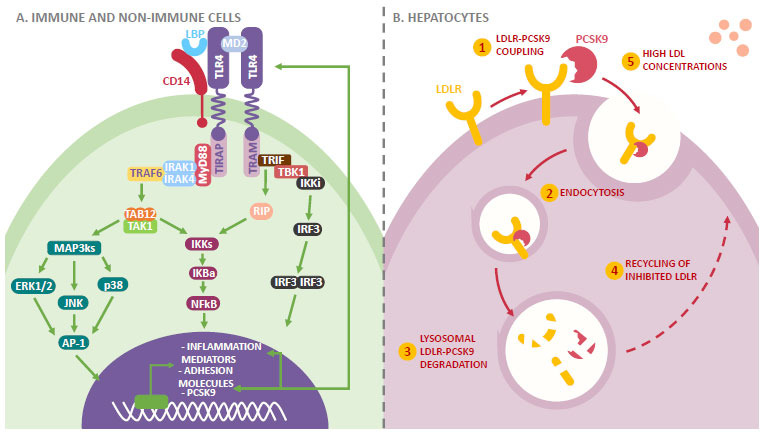
(**A**) Endotoxemia: inflammation and deregulation in cholesterol metabolism. High LPS concentrations can activate intracellular pathways that lead to the activation of transcription factors, such as NF-𝜅B and AP-1. These contribute to the induction of pro-inflammatory mediators and adhesion molecules through the TLR4 present in immune and non-immune cells. In parallel to the routes triggered by LPS, a fat-rich diet increases PCSK9 expression, which can also be promoted by the coupling of LPS to its receptor, favoring even more the pro-atherogenic inflammatory response. In addition, the cytokines involved in the inflammatory mechanism led by LPS also regulate PCSK9 expression. This adds another layer of metabolic interaction between both of them. (**B**) On the other hand, the union of the catalytic domain of PCSK9 with the EGF-A domain of the LFLR located in the cellular membranes of the hepatocytes trigger the endosomal process. This leads to the degradation of LDLR and impairs their recycling, which prevents the capture of circulating LDL, indirectly increasing the plasma levels of this molecule. This leads to higher cardiovascular risk for the individual. **Abbreviations:** TLR4: toll-like receptor 4; LBP: lipopolysaccharides binding protein; CD14: cluster of differentiation 14; MD2: myeloid differentiation protein 2; TIRAP: TIR domain-containing adaptor protein; TRAM: TRIF-related adaptor molecule; IRAK: IL-1R associated kinase; MyD88: myeloid differentiation primary response 88; TRAF6: tumor necrosis factor receptor-associated factor 6; TAB2/3: TAK2/3 binding protein; TAK1: transforming growth factor-β-activated kinase 1; MAPks: MAP kinase kinase; ERK1/2: Extracellular signal-regulated pro-tein kinases 1 and 2; JNK: c-Jun N-terminal kinases; AP-1 activator protein-1; IKKs: IkB kinase complex; NFkB: 𝜅B nuclear factor; RIP receptor-interacting protein 1; TRIF: TIR-domain-containing adapter-inducing interferon-β; TBK1: TANK-binding kinase 1; IRF3: interferon regulatory factor 3; PCSK9: proprotein convertase subtilisin/kexin type 9 serine protease; LDL: low-density lipoprotein; LDLR: LDL receptor.

At high concentrations, LPS can activate intracellular pathways, which leads to the activation of transcription factors that include the ĸB nuclear factor (NF-ĸB) and the activating protein 1 (AP-1). These contribute to the induction of proinflammatory mediators in macrophages through the Toll-like receptors 4 (TLR4) pathway (Fig. **1A**) [17]. TLR4 belong to the pattern recognition receptors (PRRs) family, and they are formed by an extracellular domain of 608 residues and an intracellular domain of 187 residues. These are involved in the intracellular signaling cascade [18].

On their own, TLR4 are not sufficient for LPS recognition. Therefore, they need to couple with the myeloid differentiation protein 2 (MD2) on the cellular surface for the ligand-induced activation of the response. MD2 lacks transmembrane and intracellular domains, which is why it has a non-covalent association with the extracellular domain of TLR4 through interaction with LPS. This results in the formation of the TLR4/MD2 receptor complex [19]. Other accessory molecules, such as LPS binding proteins (LBP) and CD14, are also capable of favoring LPS detection and serving as LPS chaperones to the TLR4-MD2 signaling complex [20].

Once the union with the LPS takes place, the dimerization of the TLR4/MD2 complex occurs. This results in conformational changes in the TLR4 homodimer. These changes lead to the recruitment of adaptive proteins that have Toll/interleukin-1 receptor (TIR) domains. Four adaptive proteins have been identified so far. The 1) myeloid differentiation primary response protein (MyD88), 2) MyD88-adapter-like protein (MAL), 3) TIR-domain-containing adapter-inducing interferon-β (TRIF), and 4) the TRIF-related adaptor molecule (TRAM). These proteins participate in two different intracellular signaling pathways. One is a direct pathway dependent on MyD88, which induces the production of proinflammatory cytokines. The second one is a pathway independent of MyD88, which induces the production of type I interferon (INF) [21].

The MyD88-dependent pathway takes place mainly on the plasmatic membrane, and it involves the fast recruitment of MyD88 and MAL proteins. These adaptor molecules stimulate the recruitment and phosphorylation activation of IL-1R-associated kinases (IRAK) [22]. IRAK (IRAK1, IRAK2, and IRAK4) recruit the tumor necrosis factor receptor (TNFR)-associated factor 6 (TRAF6). This leads to the activation of the transforming growth factor-β-activated kinase 1 (TAK1) mediated by adaptive proteins, TAK1-binding protein 2 and the TAK1-binding protein 2 (TAB2 and TAB3). TAK1 leads to the MAP-kinase-kinase (MAPKK)-mediated activation of the mitogen-activated protein kinases (MAPK). These are the c-Jun N-terminal kinases (JNKs), p38, extracellular-regulated kinases (ERK1/2), and the IkB kinase complex (IKK). This leads to the transcription of different factors, such as the nuclear factor kappa-light-chain-enhancer of activated B cells (NF-ĸB) and the activator protein 1 AP-1, which promote the production of proinflammatory cytokines [21].

The activation of the pathway independent of MyD88 takes place in the endosomal compartment after the internalization of the TLR4-MD2 complex, and it involves the recruitment of the adaptor proteins TRIF and TRAM. TRIF induces the formation of the receptor-interactive protein (RIP). This is an important component for the activation of the NF-ĸB. Furthermore, TRIF and TRAM promote the activation of TRAF3, which induces the recruitment of the threonine/serine kinase 1 (TRFK1) and IKKs. This is followed by the activation and translocation in the nucleus of the IFN regulatory factor 3 (IRF3), which promotes the production of IFN type 1 [23, 24].

It is important to highlight that TLR4 are expressed on the surface of non-immune cells (epithelial cells, endothelial cells, and fibroblasts) and immune cells (B and T cells, dendritic cells, and macrophages). The latter are the ones responsible for the critical role TLR4 play in the inflammatory process [25]. LPS integrates with the LPS binding proteins present in macrophages, transporting stimulus signals to the macrophages through the lipid bilayers present in endothelial membranes through the action of TLR4. Macrophages play a key role in the development of inflammation by releasing different proinflammatory mediators. These include TNF-α, interleukins 6 and 8 (IL-6 and IL-8), cyclooxygenase-2 (COX-2), inducible nitric oxide synthase (iNOS), and chemokines, such as the monocyte chemoattracting protein (MCP-1), the vascular endothelial growth factor (VEGF), intercellular cell adhesion molecules (ICAM-1), and the vascular cell adhesion molecule (VCAM-1) [26, 27].

Likewise, it has been observed that LPS affects the vascular smooth muscle cell (VSMC) through TLR4/Ras-related C3 botulinum toxin substrate 1 (Rac1)/protein kinase B (Akt) (TLR4/Rasc1/Akt) signaling [28]. These cells promote an environment primed for inflammation of the vascular walls through the release of pro-inflammatory cytokines and the retention of modified lipoproteins, as well as the formation of a fibrous layer that makes the plaque stable [29]. The resulting inflammation, as well as the adhesion of monocytes and cholesterol accumulation, can lead to severe endothelial dysfunction. This results in the formation of foamy cells with the subsequent development of atherosclerotic plaque [30].

Parallel to the previously described pathways triggered by LPS, there are associated signaling cascades that perpetuate the inflammatory cycle. On one hand, high-fat diets increase the expression of the proprotein convertase subtilisin/kexin type 9 (PCSK9). On the other hand, LPS translocation and its coupling with TLR4 stimulate PCDK9 transcription, which increases TLR4 transcription and favors the previously explained inflammatory response [31]. In addition to this, cytokines involved in the inflammatory mechanism led by LPS are also regulators of the expression of PCDK9, which adds another layer of metabolic interaction (Fig. **1A**) [32].

PCSK9 is an important regulating enzyme in the metabolism of cholesterol that has been identified as a risk factor for atherosclerosis, coronary events, and hypercholesterolemia [33-36]. PCSK9 is present in different tissues of the body, but its expression is highest in hepatocytes [37]. The union of the PCSK9 catalytic domain with the EGF-A domain of the low-density lipoprotein (LDL) receptors (LDLR) located on the cell membranes of the hepatocytes triggers the endosomal process. This leads to the degradation of the LDLR, preventing its recycling, which, as a consequence, impairs the capture of circulating LDL particles. As a consequence, the blood levels of this protein increase indirectly, putting the individual at a higher risk of CVD (Fig. **1B**) [38-40].

#### 1.1.2 Diet and Microbial Metabolites - direct Mechanisms

Numerous recent studies point out the influence of diet precursors on the GM and the significant impact of microbe metabolites derived from the diet, such as bile acids (BA), trimethylamine N-oxide (TMAO), and SCFA. These mainly favor a state of hypercholesterolemia and inflammation, contributing to the development of atherosclerosis [41].

##### 1.1.2.1 Bile Acids Signaling Disruption

BA are synthesized in the liver through reactions caused by cytochromes P450s (CYP) and the cholesterol 7α-hydroxylase (CYP7A1), which cause cholesterol oxidation [42]. Afterward, the oxidized cholesterol conjugates with glycine or taurine to form the primary BA, which are cholic acid (CA) and chenodeoxycholic acid (CDCA). These are stored in the gallbladder and, during the postprandial period, they are released in the duodenum [43]. Conjugated BA can be reabsorbed in the ileum and taken back to the liver through the enterohepatic circulation, preserving more than 95% of the BA pool [44]. However, a portion of these BA is usually susceptible to GM modifications through the biliary salts hydrolase (BSH), which hydrolyzes and deconjugates glycine or taurine from the primary BA. Once they are no longer conjugated, the BA can be metabolized by microbial mechanisms, such as 7 α-dehydroxylation, dehydrogenation, and epimerization. This results in the production of secondary BA, such as deoxycholic acid (DCA) and lithocholic acid (LCA) [45], which are hydrophobic components that facilitate the fecal excretion of about 5% of the BA [44].

Recently, it has been observed that BA act as signaling molecules in multiple processes through their interaction with receptors, such as the farnesoid X receptor (FXR). This has an important role in controlling the expression of genes involved in the metabolism of BA, lipids and carbohydrates, metabolism expenditure, and inflammation [46, 47].

**Fig. (2):**
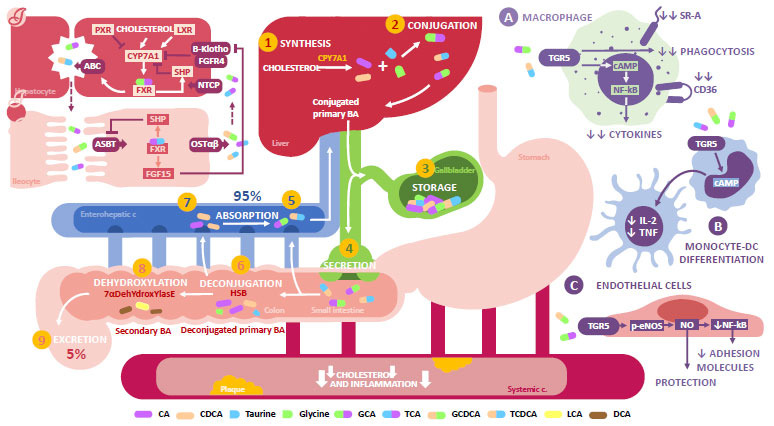
Bile Acids metabolism and its role in atherosclerosis. Primary BA (1) are synthesized in the liver from cholesterol thanks to the action of the CYP7A1. BA conjugate (2) with glycine or taurine to form conjugated BA, which (3) are stored in the gallbladder. After a meal (4), they are released inTO the duodenum. Once in the gut, BA can be (5) directly absorbed in the ileum or (6) be deconjugated through the action of bacteria with BSH ac-tivity present in the colon (7) where they ARE absorbed. This way, 95% of the BA pool is preserved while (8) the remaining BA suffer a dihydroxylation process mediated by GM, forming secondary BA. These are hydrophobic components that facilitate fecal excretion of around 5% of BA. BA also serve as a FXR ligand that, when activated at the hepatic level (I), favors their secretion by promoting SHP transcription. This inhibits CYPA71, leading to a negative feedback loop. In the enterocyte (II), SHP transcrip-tion inhibits BA reabsorption, increasing the excretion of BA. Likewise, in the ileocyte, FXR activation promotes FGF15/19 tran-scription, which acts on the FGFR4/b-klotho in the hepatocyte. This leads to the repression of CYP7A1 expression and the maintenance of BA homeostasis. Lastly, BA can also act on the TGR5, which is expressed on immune and non-immune cells. More specifically, TGR5 activation (**A**) in macrophages appears to mitigate their activation and phagocytic activity, the produc-tion of pro-inflammatory cytokines, and the expression of CD36 and SR-A. Similarly, it promotes (**B**) the differentiation of mon-ocytes to DCs, and (**C**) it mediates eNOs phosphorylation and the consequent production of NO. In addition, it inhibits the mon-ocyte adhesion process in response to inflammatory stimuli. BA have an atheroprotective role through the metabolism of cho-lesterol and the regulation of inflammation and endothelial function. This role can be disrupted in a state of dysbiosis. **Abbreviations:** PXR: pregnane X receptor; LXR: liver X receptor; CYP7A1: cholesterol 7α-hydroxylase; FXR: farneosoid X receptor; SHP: small heterodimer partner; Β-Klotho: subfamily β of Klotho protein; FGFR4: fibroblast growth factor receptor 4; NTCP: sodium ion/bile acid cotransporter; ASBT: apical sodium Ddpendent bile acid transporter; FGF15: fibroblasts growth factor 15; OSTαβ: organic solute transporter αβ; BA: bile acids; HSB: bile salts hydrolase; TGR5: G-protein-coupled bile acid receptor; SRA: scaven-ger receptors class A; CD36: differentiation cluster 36; cAMP: Cyclic adenosine monophosphate; FNkB: 𝜅B nuclear factor; IL: in-terleukin; TNF: tumoral necrosis factor; DC: dendritic cells; eNOS: endothelial nitric oxide synthase; CA: cholic acid; CDCA: Chenodeoxycholic acid; GCA: glycocholic acid; TCA: taurocholic acid; GCDCA: glycochenodeoxycholic Acid; TCDCA: tau-rochenodeoxycholic acid; LCA: lithocholic acid; DCA: deoxycholic acid.

The discovery of BA as FXR ligands has led to the identification of their function in the enterohepatic recycling of BA and the regulation of the BA feedback synthesis both in the liver and the gut [48]. The activation of hepatic FXR stimulates the secretion of BA in the gut, promoting the nuclear transcription of the small heterodimer partner (SHP), which inhibits the transcription of the CYP7A1 gene. This results in a negative feedback mechanism in the synthesis of BA [49, 50]. SHP also inhibits the reabsorption of BA in the gut, increasing its excretion [51]. In the case of FXR activation in the ileum enterocytes, FXR leads to the transcription of the fibroblast growth factor (FGF15/19), which is secreted in the portal vein and transported to the liver. There, it couples with the fibroblast growth factor receptor 4/beta klotho (FGFR4/b-klotho), activating the ERK1/2 signaling cascade and repressing CYP7A1 expression [52]. Therefore, FXR controls the expression of different protein genes and maintains BA homeostasis (Fig. **2**).

SBH levels decrease in the presence of GD, slowing down the conversion of primary BA to secondary BA. This leads to greater BA reabsorption in the enterohepatic circulation, an increase in FXR activation in the liver, and the inhibition of CYP7A1 and the liver X receptor (LXR) [53]. In normal conditions, LXR leads to positive regulation of the transporters that belong to the ATP-binding cassette subfamily G (ABCG5/ABCG8). These promote the decrease of cholesterol gut absorption in the gut and facilitate its biliary excretion. This way, LXR inhibition also results in the down-regulation of ABCG5/ABCG8 [54]. Therefore, SBH decrease due to GD can lead to increased activation of FXR, which in turn decreases BA production through its different signaling pathways. This favors the accumulation of cholesterol and could lead to the formation of foamy cells and atherosclerotic plaque [53].

On the other hand, the G-protein-coupled bile acid receptor, Gpbar1(TGR5), is expressed in different tissues, such as the gut and the liver, and in specific cells, like macrophages and endothelial cells. It has protective functions at different levels, influencing the composition and homeostasis of BA and decreasing the expression of adhesive molecules in the endothelium, as well as inhibiting the production of proinflammatory cytokines in macrophages (Fig. **2**) [55]. It has been observed that BA are capable of inhibiting the secretion of TNF-α in human THP-1 monocytes with overexpression of TGR5 [56]. In animal models, agonists of this receptor inhibit the activation of macrophages and the production of TNF-α, possibly through the cAMP-dependent inhibition of the NF-ĸB [57]. Inhibition of the NF-ĸB has demonstrated a modulating role in the accumulation of lipids in macrophages and the reduction of the formation of foamy cells through up-regulation of PPAR, LXR, and ABCA1, countering the development of atherosclerosis [58].

Pols *et al.* demonstrated that TGR5 activation has a protective role in the development of atherosclerosis in LDLr-/- mice receiving a cholesterol-rich diet. Specifically, its activation in macrophages was able to inhibit inflammation through the TGR5-cAMP-NF-ĸB pathway. Similarly, it reduced the expression of CD36 and SR-A, two receptors involved in the endocytosis of LDL-ox. Together with the inhibition of NF-ĸB, this can contribute to decreasing the formation of foamy cells. These results coincided with the decrease in inflammation and concentration of intracellular macrophages, resulting in a more stable phenotype and suggesting a lower risk of rupture [57]. In addition to this, TGR5 activation appears to favor Akt-dependent eNOS phosphorylation and the concentration of Ca^2+^, increasing NO production and inhibiting the monocyte adhesion process in response to inflammatory stimuli [59].

##### 1.1.2.2 Role of the Trimethylamine N-oxide Metabolism

The pro-atherogenic role of GM through TMAO metabolism has been recently demonstrated. TMAO is a metabolite derived from trimethylamine (TMA), and it has been linked with direct processes that lead to the development of atherosclerosis, inflammation, and platelet hyperreactivity [60].

TMA is mainly generated from diets with a high amount of L-carnitine and choline. L-carnitine is mainly present in red meats and dairy products [61], while choline (free or/and esterified), like lecithin or phosphatidylcholine (PC), is found in foods, like egg yolk, the liver, meat, high-fat dairy products, and some nuts and beans [62]. L-carnitine can transform into TMA through the direct action of the carnitine oxidoreductase enzyme. An indirect pathway is through the carnitine-CoA transferase and/or carnitine-TMA-lyase, which first transform L-carnitine into γ-butyrobetaine. Then, this is converted into TMA through the carnitine-TMA-lyase [62, 63]. On the other hand, choline can convert into TMA directly through the action of the choline-TMA-lyase, or it can be oxidized to betaine through the choline dehydrogenase and the betaine aldehyde dehydrogenase. Afterward, it is transformed into TMA through the action of betaine reductase [64]. This series of enzymatic reactions are mediated by the GM and, once it is produced, TMA is absorbed and reaches the hepatocytes through the hepatic vein. There, TMA is metabolized by flavin monooxygenases 1 and 3 (FMO1 and FMO3) to generate TMAO [64]. Therefore, L-carnitine and choline metabolization to generate TMAO do not only depend on their consumption but also the balance and diversity of the GM.

The GM has a role in the degradation of L-carnitine through the cleavage of the 3-hydroperoxide, which is regulated by the responsible microbes, such as Proteobacteria and Bacteroidetes. In addition, the GM also participates in the degradation of choline through the division of the carbon-nitrogen link in its structure. This process is mainly regulated by the Firmicutes and Proteobacteria phylum and six microbial genera, which are Anaerococcus hydrogealis, Clostridium asparagiforme, Clostridium hathewayi, Clostridium sporogenes, Escherichia fergusonii, Proteus penneri, Providencia rettgeri, and Edwardsiella tarda [65, 66].

**Fig. (3):**
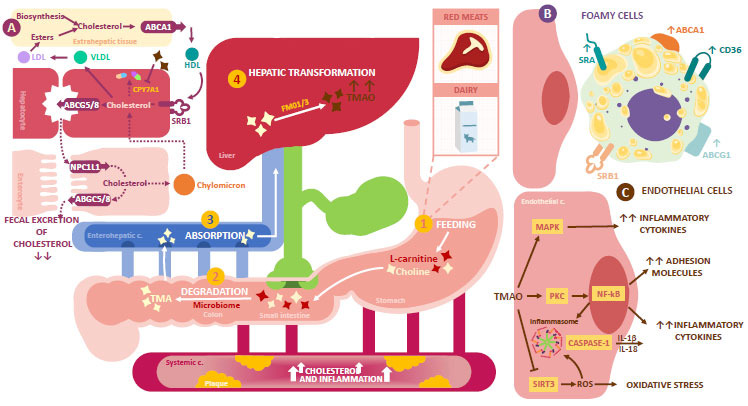
TMAO metabolism and its role in atherosclerosis. (1) Red meat and dairy ingestion are the main source of L-carnitine and choline, two metabolites that (2), once degraded in the colon through the action of the GM, are transformed into TMA. (3) Once TMA is formed, it enters the enterohepatic circulation (4) where it is metabolized by the FMO1/3 to generate TMAO at the hepatic level. The production of TMAO increases in the presence of dysbiosis. Increased TMAO levels can favor atherosclerosis through different mechanisms. Initially, they can (**A**) inhibit RTC by interfering with BA synthesis and CPY7A1 expression. At the same time, increased TMAO levels can down-regulate the ex-pression of cholesterol absorption targets, such as NPC1L1 and ABCG5/8. This leads to extremely low concentrations of the ef-flux of cholesterol from the liver to the bile and the feces. Likewise, high TMAO levels promote the (**B**) up-regulation of macro-phages and their transformation into foamy cells by increasing SR-A, CD36, ABCA1, and ABCG1 expression. Lastly, this metabo-lite is also able to act on (**C**) endothelial cells, increasing the production of pro-inflammatory cytokines, adhesion molecules, and oxidative stress. This generates an environment prime for the development of atherosclerosis. **Abbreviations:** CYP7A1: choles-terol 7α-hydroxylase; FNkB: 𝜅B nuclear factor; ABCA: ATP binding cassette transporter; ABGC: ATP Binding Cassette Subfamily G; LDL: low-density lipoprotein; VLDL: very low-density lipoprotein; HDL: high-density lipoprotein; NPC1L1: Niemann-Pick C1-Like 1; FMO: flavin-containing monooxygenase; TMAO: trimethylamine N-oxide; MAPK: mitogen-activated protein kinase; PKC: protein kinase C; ROS: reactive oxygen species; SIRT3: sirtuin 3; SRA: scavenger receptors class A; SRB1: scavenger recep-tors class B type 1.

GD can then directly lead to increased TMAO levels and the development of atherosclerosis [67]. This would take place through two mechanisms, the first one being the induction of endothelial inflammatory damage. The second one corresponds to alterations in cholesterol metabolism due to the inhibition of reverse cholesterol transport (RCT), the up-regulation of macrophages expression, the down-regulation of the expression of absorption and cholesterol targets [68], and the reduction of CYP expression (Fig. **3**) [69].

### 1.2 Endothelial Inflammatory Damage

Studies involving mice fed a Western diet have shown higher levels of plasma TMAO as well as higher expression of the proinflammatory cytokines, TNF-α and IL-1β. In addition, a decrease in the expression of the anti-inflammatory cytokine IL-10 has also been observed. This promotes vascular inflammation and oxidative stress, contributing to endothelial damage [70-72]. A clinical study involving 81 patients with stable angina suggested increased blood levels of TMAO to be significantly associated with IL-1β and high-sensitivity C-reactive protein (hs-CRP), as well as with an increase in inflammation and decrease in cultivated endothelial progenitor cells (EPC). This study also showed that the incubation of the TMAO in EPC led to cellular inflammation and an increase in oxidative stress, eventually leading to endothelial dysfunction [73].

*In vivo* and *in vitro* studies have shown that TMAO can worsen inflammation by activating MAPK and NF-κB signaling pathways, which leads to the induction of inflammatory proteins, such as L-6, COX2, E-selectin, and VCAM-1. This leads to leukocyte adhesion to endothelial cells and the decrease of endothelial auto-repair processes, accelerating endothelial dysfunction and promoting the early pathological process of atherosclerosis [74, 75].

### 1.3 Disruption of Cholesterol Metabolism

#### 1.3.1 Inhibition of Reverse Cholesterol Transport

Some studies have revealed that TMAO partially regulates the metabolism of cholesterol and sterol as well as their enterohepatic circulation through different mechanisms, including the synthesis and secretion mechanism of BA [76]. Specifically, TMAO decreases the synthesis of BA and the hepatic BA transporters, causing an effective reduction of the BA complex, suppressing RCT, and decreasing cholesterol absorption by enterocytes. Likewise, it has been observed that choline supplementation, carnitine administration through the diet, or even direct TMAO supplementation could promote RCT suppression [77, 78]. Since RCT involves the main function of countering the deposition of excess cholesterol in the peripheral tissues, it is considered atheroprotective in non-sclerotic stages. However, when this mechanism is inhibited, hypercholesterolemia worsens, leading to the accumulation of cholesterol in the vascular wall and favoring the development of atherosclerotic plaques [79].

#### 1.3.2 Up-regulation of Macrophage Expression

In atherosclerosis, LDLs in the vascular walls tend to oxidize, transforming into modified LDL (ox-LDL). It has been recently described that TMAO increases the expression of scavenger receptors (SR), CD36 and SR-A1, present in macrophages and with a high affinity to detect and ingest ox-LDL [65].

The class of SR-A (SR-A1 and A2) expressed on the surface of macrophages explains the absorption of acetylated LDL in the majority of macrophages. However, these cells have a higher affinity for ox-LDL, recognizing the ApoB-modified components [80]. Likewise, studies have demonstrated the mediating role of the MAPK/JNK pathway in the trapping of ox-LDL related to CD36. It has been observed that when the MAPK inhibitor SB230580 and the JNK inhibitor SP600125 were used, there was a decrease in the formation of foamy cells and CD36 expression [81]. Therefore, TMAO would participate in the pathophysiology of atherosclerosis by promoting the migration of macrophages as well as the transformation of macrophages into foamy cells [77, 82].

#### 1.3.3 Down-regulation of Cholesterol Absorption Targets

One of the main transporters involved in the absorption of cholesterol is the Niemann-Pick C1-Like 1 (NPC1L1). This is a transmembrane protein located on the apical membrane of enterocytes and the canalicular membrane of hepatocytes. Its function is to transport non-esterified cholesterol from the gut lumen to the enterocytes [83]. Once inside the enterocyte, the cholesterol is esterified and packaged in chylomicrons to be secreted to the lymph and transported to the liver through enterohepatic circulation. In the liver, the NPC1LI on the membrane of the hepatocytes facilitate the absorption of biliary cholesterol to the inside of these cells. Other transporters involved are ABCG5 and ABCG8. These are also present on the apical membrane of hepatocytes and enterocytes, limiting intestinal absorption and facilitating biliary secretion of cholesterol [84].

TMAO diet supplementation significantly decreased the expression of both gut cholesterol transporters (NPC1L1 and ABCG5/8), decreasing cholesterol absorption and its metabolism [61]. In the case of NPC1L1, its decrease is associated with the interruption of the enterohepatic circulation of cholesterol, causing an important loss of exogenous cholesterol. Therefore, endogenous cholesterol decreases, leading to positive feedback for endogenous cholesterol synthesis [85]. Regarding the decrease of the hepatic expression of ABCG5 and ABCG8, mice studies have demonstrated that it causes the attenuation of the RCT. This leads to extremely low concentrations of the exit flow of cholesterol from the liver to the bile and feces, which could contribute to the development of atherosclerosis [86].

#### 1.3.4 Decrease in CYP Expression

TMAO also appears to affect cholesterol metabolism in the liver, where it has an important role of affecting the BA metabolic synthesis pathway. This is a key element in the elimination of excess cholesterol from blood circulation [87]. A mice study performed on ApoE-/- mice demonstrated that TMAO mediated FXR and SHP activation, which caused the inhibition of CYP7A1 expression in the classic BA synthesis pathway. Therefore, the hepatic synthesis of BA was repressed, accelerating the formation of aortic atherosclerosis [60]. In another study, the mice received a diet supplemented with TMAO. The researchers observed a significant decrease in cholesterol absorption as well as in the hepatic expression of CYP7A1 and CYP21A1 [61].

It has been demonstrated that changes in GM composition can increase TMAO plasma production. This results in different physiopathological mechanisms that involve the disruption of the BA signaling pathways as well as in cholesterol and sterol metabolism. In addition, different inflammatory signaling pathways are activated, which eventually leads to endothelial dysfunction and atherosclerosis [60].

#### 1.3.5 Decrease in the Production of SCFA

SCFA are the main metabolites produced in the colon through bacterial fermentation. The substrates come from fiber-rich diets and resistant starches [88]. SCFA have from one to six carbon atoms, including acetate (c2), propionate (C3), and butyrate (C4), and they are locally used as energy sources by enterocytes. They are also transported to the blood circulation, where they can interact with peripheral cells and tissues [16, 89].

SCFA appear to have a role in inflammation regulation by mediating the suppression or activation of immune cells, the expression of cytokines, and the induction of apoptosis. In the latter, their regulating effect can result in a protective phenomenon or in the promotion of atherosclerosis [90]. These functions take place through the inhibition of the histone deacetylases (HDACs) and/or the activation of G protein-coupled receptors (GPRs), including GPR4 (or free fatty acid receptor 2, FFA2) and GPR41 (or FFA3) [91].

GPR41 plays an important role in the regulation of the immune and inflammatory responses because it is mainly expressed by immune cells, such as neutrophils, monocytes, and dendritic cells. This differs from GPR43, which has less expression in these cell types, with a greater presence in the fatty tissue, pancreas, and endothelial cells [92, 93]. The three GPRs can be activated by the different SCFA; however, their specificity, potency, and regulating action vary according to the ligand and cell involved. This way, propionate and butyrate are more potent than acetate for GPR41 activation, while propionate and acetate are more potent than butyrate for GPR43 activation [91].

HDACs oppose the function of histone acetyltransferases, mediating the elimination of acetyl groups from lysine residues present in histones. Therefore, their inhibition increases their acetylation, decreasing their positive charge and their capacity to couple with DNA. In consequence, HDACs inhibition results in chromatin expansion and gene transcription, including those involved in the immune response, vascular integrity, and the development of cardiovascular disease (CVD) [94]. Both butyrate and propionate are non-competitive inhibitors of HDACs [95, 96], while the inhibitory capacity of acetate is still debated [97, 98].

**Fig. (4):**
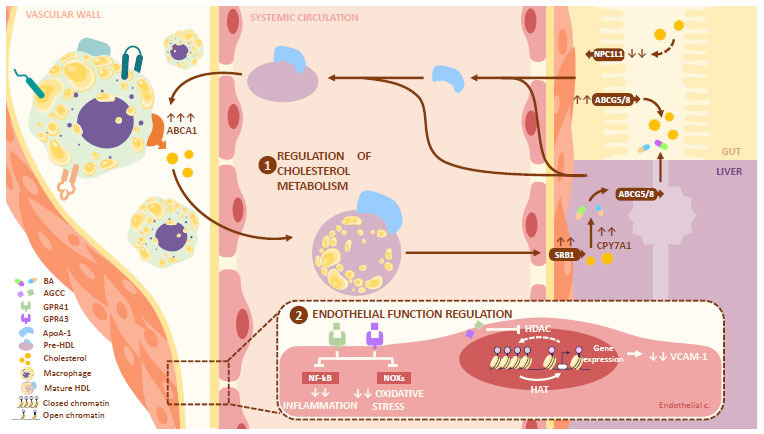
Role of SCFA in atherosclerosis: Among the variety of SCFA, the one with the highest evidence regarding its protective role in atherosclerosis is butyrate. More specifically, butyrate appears to be involved in the different steps of cholesterol metabolism, including gut absorption and elimination of excess cholesterol as BA. It does this by favoring the up-regulation of CYP7A1, ABCA1, ABCG5/8, SRB1, and the down-regulation of NPC1L1. Similarly, SCFA can attenuate inflammation, oxidative stress, and VCAM expression induced by TNFα; and increase NO bioavailability, regulating endothelial function. **Abbreviations:** ABC ATP binding cassette transporter; NPC1L1: Niemann-Pick C1-Like 1; ABCG: ATP Binding Cassette Subfamily G; CPY7A1: cholesterol 7α-hydroxylase; SRB1: scavenger receptors class B type 1; BA: bile acids; SCFA: short-chain fatty acids; GPR: G protein-coupled receptors; HDL: high-density lipoprotein; HDAC: histone deacetylases; HAT: histone acetyltransferases; VCAM-1: vascular cell adhesion molecule 1; NFkB: 𝜅B nuclear factor; NOXs: nitrogen oxides.

Although diet and GM composition can influence SCFA synthesis [99], controlled studies involving mice exposed to diets rich in fats and cholesterol have associated them with a significant decrease in SCFA levels. The largest decrease is seen in butyrate concentrations [100, 101]. Interestingly, among the different SCFA, butyrate has shown the greatest evidence of having a protective role against atherogenesis [91, 102]. Among the proposed mechanisms, there are 1) the regulation of endothelial dysfunction and inflammation, as well as 2) the regulation of cholesterol metabolism (Fig. **4**).

### 1.4 Regulation of Endothelial Dysfunction

Both LPS and circulating pro-inflammatory cytokines can induce endothelial activation. This is characterized by an increase in the production of anti-inflammatory and adhesion molecules, which are key elements in the development of atherosclerosis. Preclinical studies have reported that the administration of SCFA can significantly inhibit the production of IL-6 and IL-8. This decrease has been attributed to the action of acetate on the GPR41/GPR43 and the inhibitory function of butyrate and propionate on HDACs [91].

Likewise, it has been reported that butyrate and propionate decrease VCAM-1 expression induced by TNF-α. This leads to lower adhesion of monocytes to the endothelium, impairing the formation of foamy cells [91, 103, 104]. Interestingly, the expression of this molecule in endothelial cells stimulated by TNF-α appears to be regulated by the process of intracellular acetylation. Similarly, the knockdown of the siRNA of HDAC3 in human umbilical vein endothelial cells (HUVECs) decreases the expression of VCAM-1 [91]. Together with the knowledge that the inhibitory effects of butyrate and propionate on the expression of this molecule are not mediated by GPRs, it can be inferred that they are the result of HDAC3 inhibition by these SCFA [91, 103, 104].

A study involving ApoE-/- mice showed a decrease in endothelial dysfunction and migration of macrophages in atherosclerotic lesions through the reduction of oxidative stress after butyrate exposure [105]. Similarly, it has been reported that butyrate and acetate can counter endothelial dysfunction induced by angiotensin II by increasing the bioavailability of nitric oxide through a GPR41/43 pathway [105]. Thanks to its antioxidant role, butyrate has shown an anti-atherogenic role in mice endothelial cells by inhibiting the activation of the NLRP3 inflammasome and, in consequence, the formation of the neointima [106].

### 1.5 Regulation of Cholesterol Metabolism

According to preclinical studies, butyrate also appears to be involved in the different steps of cholesterol metabolism. These include gut absorption and elimination of excess cholesterol as BA. This takes place because butyrate favors the up-regulation of CYP7A1, ABCA1, and ABCG5/8, and down-regulation of NPC1L1 [107, 108]. Likewise, butyrate is capable of decreasing the synthesis and oxidation of FA in the liver by decreasing the expression of CYP4A14 and Acot1/2, which is responsible for the synthesis of acyl-CoA thioesterases 1 and 2 [108].

Likewise, sodium butyrate appears to induce the transcriptional activity of SR-B1 (class B, type 1) in HepG2 cells. SR-B1 plays an important role in cholesterol efflux. Its up-regulation in mice results in a decrease in the concentration of cholesterol esters in HDL and a lower risk of atherosclerosis [109]. Furthermore, the exposition of ApoE-/- to butyrate led to an increase in the expression and activity of the ABCA-1 transporter and the efflux of cholesterol in macrophages, countering the atherosclerotic process [108]. At the same time, propionate and butyrate reduce the proinflammatory responses mediated by 1NF-kB, increasing the transcription of ApoA-1 in inflamed HepG2 cells [110]. This provides additional evidence of the role of butyrate in RCT.

#### 1.5.1 Phenylacetylglutamine

Phenylalanine is an essential amino acid present in protein-rich foods, which is absorbed in the small intestine and can be metabolized into phenylpyruvic acid and then into phenylacetic acid (PAA) through the action of GM in the colon, where it is absorbed. PAA is quickly transformed into phenylacetylglutamine (PAG1n) in the liver, a metabolite recently correlated with the development of CVD and its main adverse complications (MACE) in patients with and without DM [111].

It is important to highlight that the mechanisms involved are still uncertain. However, Nemet *et al.* performed a series of *in vitro* and *in vivo* assays, and they proposed that PAGln can modulate platelet function by favoring a prothrombotic phenotype. These effects were attributed to the union of this metabolite to GPCRs in the adrenergic receptors (ADRs) A2A, A2B, and β. These receptors are expressed in platelets and have been observed to regulate their functions in other contexts. Likewise, these authors demonstrated that the administration of carvedilol could mitigate ADRs signaling induced by PAG1n as well as *in vivo* thrombosis [111]. In addition, PAG1n would also interfere in different physiologic processes, as ADRs are widely expressed across most cell types and have an important role in vascular and myocardial function as well as in systemic metabolism and homeostasis [111].

## 2 MICROBIOTA AND ATHEROSCLEROSIS - THERAPEUTIC ASPECTS

### 2.1 Modulation of Gut Microbiome and its Metabolites

As has been previously mentioned, GM disruption can lead to the development of atherosclerosis. Therefore, the introduction of different therapeutic strategies focused on the regulation of GM and its metabolites has gained interest in the past years.

#### 2.1.1 Probiotics and Prebiotics

Probiotics are live microorganisms that, when correctly handled, have a beneficial effect on the host. Examples of probiotics are *Bifidobacterium, Lactobacillus*, and *Enterococcus*. Meanwhile, prebiotics are non-digestible carbohydrates, such as oligosaccharides and polysaccharides, which modulate the balance of the GM [112]. Studies involving probiotics and prebiotics, mainly performed in mice models [50, 68, 113-122], have shown their cardioprotective and anti-atherogenic properties. These include their antioxidant [123], antiaggregant [124], and anti-inflammatory [125, 126] activities, as well as the capacity to decrease serum lipid levels [127, 128]. This is achieved by promoting the growth of beneficial bacteria, favoring the function of the gut barrier, the production of SCFA, and the regulation of BA and cholesterol metabolism, as well as the immune response of the host (Table **1**).

#### 2.1.2 Polyphenols

Polyphenols represent a wide class of secondary metabolites of plants known for their antioxidant properties. After being ingested, around 10% is absorbed in the small intestine, while the rest of the non-absorbable polyphenols are transported to the large intestine to be catabolized by gut bacteria into absorbable metabolites. Therefore, a part of their effects depends on the GM [129].

Likewise, these catabolites can have functions on the GM, resulting in an increase in beneficial bacteria and the reduction of pathogenic bacteria, increasing the production of SCFA and promoting an anti-inflammatory response [130]. Different preclinical studies using resveratrol and quercetin have shown attenuation of cholesterol accumulation, endothelial dysfunction, inflammation, and a reduction of the area of the atherosclerotic plaque (Table **2**) [131-141].

Likewise, the relationship between different polyphenols and other vegetal complexes has been studied in the context of their effect on PCSK9 with mixed results. The main limitations correspond to bioavailability and study models that can be performed *in vivo* and in humans. Despite this, different studies have remarked on the potential of polyphenols in the regulation of PCSK9 as a complement to statin treatment, with lines of research looking into the development of solutions for these technical and biological limitations [142, 143].

#### 2.1.3 TMAO Inhibitors

Considering the critical role of TMAO in the development of CVD, different strategies targeting TMAO and its precursor TMA have been proposed (Table **2**). The use of choline analogs, such as 3,3‐dimethyl‐1‐butanol [136], fluoro-methyl choline, and iodomethylcholine [137] in mice models, has shown that inhibition of the microbial TMA lyase results in the decrease of TMAO serum levels and the reduction of the area of the plaque. A clinical trial [138] reported the use of meldonium as effective in the inhibition of γ‐butyrobetaine hydroxylase and the reduction of L-carnitine. However, this drug is not approved by the Food and Drugs Administration (FDA), and its distribution is limited to East European countries.

### 2.4 Antibiotics

Multiple randomized placebo-controlled assays have been performed to evaluate if antibiotic treatment has beneficial effects on GM and the development of CVD (Table **2**). Based on this, mice assays with neomycin and polymyxin [139], as well as with ampicillin [140], have demonstrated efficacy by reporting a decrease in the area of the plaque and atherosclerotic lesions, respectively. However, another study that included the use of ampicillin, metronidazole, neomycin, and vancomycin [141] reported unfavorable changes in which the atherosclerotic lesions increased. These discrepancies between different studies could be due to the duration of the treatment and the differences in the spectrum covered by antibiotics. Therefore, additional studies would be needed to determine the role of antibiotics in the development of atherosclerosis. However, obtaining additional evidence could not be feasible due to the risk of antibiotic resistance.

### 2.5 Fecal Microbial Transplant (FMT)

FMT is the transfer of microorganisms from a healthy donor to the gut of a patient to restore the normal function of the GM [144]. Recently, the interest in demonstrating the potential benefits of this strategy for the treatment of metabolic disorders, such as metabolic syndrome [145] and cardiovascular disease, has grown [146]. However, the main limitation to the use of this therapy is that toxins and infectious agents can also be transferred, bringing new complications for the patient [147, 148]. It is expected that additional evidence in the future will better define the best preparation, dosing, and application methods of this therapy to achieve a lower risk of complications.

## 3 INTERACTION BETWEEN GM AND HYPERLIPIDEMIA TREATMENT

Interestingly, GM has proven to have an impact on the efficacy of certain drugs. Among these, there are statins, which are the first line of treatment for hyperlipidemia and CVD [149]. More specifically, different clinical assays have shown that GM diversity as well as the concentration of some of its metabolites can predict the response to treatment [150, 151]. Similarly, both preclinical and clinical studies report that statins can modify GM composition (Table **3**) [150-159]. In this context, they appear to have a beneficial effect by reducing GD and increasing the presence of non-pathogenic bacteria. These findings suggest another mechanism through which these drugs have their effects and explain the different responses to treatment observed across individuals, as the GM varies per individual [160].

## CONCLUSION

Currently, the relationship between the multiple molecular mechanisms triggered by GD and certain types of diets is evidence of the different pathways through which they can directly or indirectly influence the development of atherosclerosis. This takes place mainly through the mediation of inflammatory processes that result in the metabolic disruptions seen in this disease. The relationship between GM and atherosclerosis has led to studies focused on these physiopathological processes in search of new therapeutic targets. This has allowed for the identification of novel alternatives in the prevention and management of atherosclerosis, with preclinical studies showing promising results. This has been especially observed in studies centered on the modulation of GM and its metabolites as well as the impact the GM has on preexisting cardiovascular treatments, such as statins. However, clinical studies are still scarce, which is why additional research is needed to evaluate the clinical potential and efficacy of these therapeutic strategies.

## Figures and Tables

**Table 1:** Use of prebiotics and probiotics in the modulation of gut microbiota in atherosclerosis.

**Authors/Refs**	**Microorganism(s)** **/Molecules**	**Study Subjects**	**Results**	**Mechanisms**
**PROBIOTICS**				
Jin Li *et al.* [113].	*Akkermansia Muciniphila*	ApoE-/- mice receiving a Western diet	↓ Area and size of the atherosclerotic lesion; ICAM-1 expression, MCP-1, IL-1β, TNF-α, and macrophage infiltration	↑ Occludin and ZO-1 expression, mucin,; ↓ endotoxemia
Rebeca Martin *et al.* [114]	*Lactobacillus rhamnosus*	C57BL/6 male mice	↓ Gut permeability and expression of IL-6, IFN-β, e IFN-γ; with ↑ IL-10	↑ GC population and mucus expression ↑ Spink-4, SLC7A7, GHR, PYY y Guca2B
Yee Kwan Chan *et al.* [115]	*Lactobacillus* *rhamnosus* GG	ApoE-/- mice receiving a high-fat diet	↓Plaque size on the aortic sinus and tree	Regulation of A-FABP expression, cholesterol, E-selectin, ICAM-1, VCAM-1 and endotoxins
Z. Guo *et al.* [116]	*Lactobacillus* or *Bifidobacterium* o *Enterococcus*	Humans	↓ TC, C-LDL y TAG ↑ C-HDL	Not established
Marta Toral *et al.* [117]	*Lactobacillus coryniformis*	C57BL/6J male mice receiving a high-fat diet	↓Gut permeability and TNF-α, IL-1β, MCP-1 levels Restoration of acetylcholine response	↑ Occludin, ZO-1 and mucin expression. ↓ Endotoxemia and TLR4 expression; ↑ NO bioavailability (↑eNOS y ↓ NOX1)
Iñaki Robles-Vera *et al.* [118]	*Bifidobacterium breve* *and Lactobacillus fermentum*	Hypertensive mice	↓ Gut permeability Prevention of SBP increase and restoration of acetylcholine response	Restoration of F/B ratio; ↑ butyrate and acetate-producing bacteria ↑ occludin and ZO-1 expression and ↓ endotoxemia; ↑ Treg y ↓Th17 ↓ NOX activity
Xi Liang *et al.* [68]	*Lactobacillus* and *Bifidobacterium*	C57BL/6 male mice reciving a choline-rich diet	↓ Serum and colon TMA and serum TMAO ↓ TC and serum TAG ↓ Hepatic accumulation of lipids	↑ Direct TMA degradation and/or GM modification (?) ↑ CYP7A1 expression and ↓ FXR expression
Matsumoto *et al.* [119]	*Bifidobacterium animalis subsp. lactis*	Healthy humans	↓ Fecal TMA and TNF-α expression	↓ TMA-producing bacteria
**PREBIOTICS**				
Chen-Jie Ling *et al.* [120]	Low-dose lactoferrin (2 mg/mL), médium dose (10 mg/mL) or high dose (20 mg/mL)	ApoE-/- mice receiving a high-fat and high-cholesterol diet	↓TC and C-LDL plasma concentration; and liver concentration of TC and FC (MD and HD). ↓ hepatic TAG (MD) ↑ Cholesterol excretion (MD and HD) ↓ Liver steatosis and area of the plaque (LD, MD and HD)	At MD ↑ gut and liver expression of ABGC5 and ↓ hepatic expression of ↓ SREBP-2, HMGCR y LDLR. At HD and LD ↑ hepatic expression of SREBP-2, LDLR and CYP7A1; and ↓ NPC1L1 gut expression
Emilie Catry *et al.* [50]	Inulin-type fructans	ApoE-/- mie receiving a diet that lacked PUFAs	Promotion of BA metabolism ↓ Endothelial dysfunction and ↑ bioavailability and NO action	↑ CYP7A1 and GLP-1 expression ↑ NO-producing bacteria; and ↓ secondary BA producing bacteria
Siddhartha Ghosh *et al.* [121]	Galacto-oligosaccharides	C57NL/6 mice receiving a Western diet	↓ Gut permeability ↓ Circulating macrophages and neutrophils	↓ Neurogenin-3 expression and ↑ number of GC ↓ Endotoxemia
Lisa R. Hoving *et al.* [122]	Manano-oligosaccharides	E3L.CETP female mice receiving a high-cholesterol diet	↓ Atherosclerotic injuries ↓ TC	↑ Bacteroidetes ↓Firmicutes; ↑ butyrate production ↑ BA fecal excretion without affecting plasma levels

**Abbreviations:** BA: bile acids; ABC: ATP-binding cassette transporters; GC: goblet cells; TC: total cholesterol; CYP7A1: cholesterol 7α-hydroxylase; C-HDL: HDL cholesterol; C-LDL: LDL cholesterol; HD: high dose; LW: low dose; MD: medium dose; eNOS: endothelial nitric oxide synthase; F/B: Firmicutes/Bacteroidetes; FC: free cholesterol; FXR: farnesoid X receptor*;* GHR: ghrelin; GLP-1: glucagon-like peptide-1; Guca2B: guanylate cyclase activator 2B; HMGCR: 3-hidroxy-3methyl-glutaryl-CoA reductase; ICAM-1: intercellular adhesion molecule-1 1; IL: interleukin; INF: interferon; GM: gut microbiome; MCP-1: monocyte chemoattractant protein-1; NPC1L1: Niemann-Pick C1-Like 1; NOX: nicotinamide adenine dinucleotide phosphate-oxidase1; PUFAs: polyunsaturated fatty acids; PYY: peptide YY; SLC7A7: solute Carrier family 7 member 7; SBP: systolic blood pressure; Spink-4: Kazal-4 protease inhibitor; TAG: triacylglycerides; TMA: trimethylamine; TMAO: trymethylamine N-oxide; TNF-α: tumoral necrosis factor-α; VCAM-1: vascular cellular adhesion molecule- 1; ZO-1: zonula occludens 1.

**Table 2:** Other strategies in the modulation of gut microbiome in atherosclerosis.

**Authors/Refs**	**Therapy**	**Study Subjects**	**Results**	**Mechanisms**
**POLYPHENOLS**				
Ming-liang Chen *et al.* [131]	Resveratrol	ApoE-/- mice	Attenuation of TMAO-induced atherosclerosis Promotion of BA metabolism	↓ Bacterial synthesis of TMA ↑ Bacteria with BSH activity, suppression of the FXR-FGF15 axis, and ↑ CYP7A1 expression
Guozhu Ye *et al.* [132]	Resveratrol	RAW 264.7 macrophages	↓ TC accumulation, esterified cholesterol, and neutral lipids	↑Cholesterol efflux mediated by the ABCA1/G1 transporter through PPAR α/γ
Jinghua Li *et al.* [133]	Resveratrol	ApoE-/- mice	↓ Endothelial dysfunction and plaque formation ↑ NO bioavailability	↑ eNOS activity through the PKA-CREB pathway
Jing Nie *et al.* [134]	Quercetin	C57BL/6 LDLR-/- mice	↓ Plaque area and size ↓ Inflammation associated with lipid and pro-atherogenic metabolites	Firmicutes and actinobacteria modulation
Qiaowen Wu *et al.* [135]	Quercetin	ApoE-/- mice and C57BL/6J mice	↓ TC, TAG, HDL y LDL ↓ Inflammation	GM modulation ↑ SCFA production and biosynthesis and expresión of BA
**INHIBITORS OF ENZYMES INVOLVED IN THE PRODUCTION OF TMAO**				
Zeneng Wang *et al.* [136]	3,3‐dimethyl‐1‐butanol	ApoE-/- mice with a choline-rich diet	↓ Plaque size ↓ Formation of foamy cells ↓ Plasma TMAO	Choline analogous and reversible inhibitor of the microbial TMA lyase Modulation of GM
Adam B. Roberts *et al.* [137]	Fluoromethylcholine and Iodomethylcholine	C57BL/6J female mice receiving a choline-rich diet	↓ Plasma TMAO ↓ Platelet aggregation and thrombi formation	Choline analogs and irreversible inhibitors of the microbial TMA lyase
Edgars Liepinsh *et al.* [138]	Meldonium	Humans	↓ L-carnitine ↓ Plasma TMAO	Suppression of the γ‐butyrobetaine hydroxylase ↓ L-carnitine reabsorption
**ANTIBIOTICS**				
Siddhartha S. Ghosh *et al.* [139]	Neomycin and Polymyxin	ApoE-/- mice receiving a Western diet	↓ Endotoxemia ↓ Plaque area	↓ Gut permeability ↑ IAP activity, and restoration of the claudin-1 and ZO-1 expression
Ida Rune *et al.* [140]	AmpicilinE	ApoE-/- mice receiving a cholesterol-rich gluten-free diet	↓ Atherosclerotic lesions	Changes in GM composition ↓ C-LDL and C-VLDL
Ben Arpad Kappel *et al.* [141]	Ampiciline, Metronidazole Neomycin Vancomycin	ApoE-/- male mice receiving normal chow or Western diet	↑ Atherosclerotic lesions	Changes in GM composition (↓ Bacteroidetes and Clostridia), disrupting different metabolic pathways (BA and tryptophane metabolism)

**Abbreviations:** BA: bile acids; ABC: ATP-binding cassette transporters; SCFA: short-chain fatty acids; BSH: bile salts hydrolase; TC: total cholesterol; CREB: cAMP-response element-binding protein; CYP7A1: cholesterol 7α-hydroxylase; C-LDL: LDL cholesterol; C-VLDL: VLDL cholesterol; FGF15: fibroblast growth factor 15; FXR: farnesoid X receptor; IAP: intestinal alkaline phosphatase; GM: gut microbiome; NO: nitric oxide; PKA: protein kinase A; PPARα/γ: peroxisome proliferator-activated receptors α/γ; TMA: trimethylamine; TMAO: trimethylamine N-oxide TNF-α: tumoral necrosis factor α; ZO-1: zonula occludens 1.

**Table 3:** Interaction between gut microbiome and hyperlipidemia treatment.

**Authors/Refs**	**Dose ** **(Duration)**	**Study Subjects**	**GM Changes**	**Effect of GM on the** ** Response to Treatment**	**Possible ** **Mechanisms**
	**ATORVASTATIN**				
Khan *et al.* [154]	20 mg/d (during the two previous years)	Patients with hypercholesterolemia	↓ Pro-inflammatory bacteria and taxons associated with the development of atherosclerosis and CVD progression; ↑ beneficial bacteria	Not established	↓ Secondary BA production
Zimmer-mann *et al.* [155]	10 mg/Kg/d (4 wk)	C57BL/6 mice	Restoration of F/B ratio	Effect of the TTM ↓ in mice with depleted GM	Regulation of the expression of genes involved in cholesterol metabolism
Sun *et al.* [151]	20 mg/d (3 months)	Patients with hyperlipidemia	Not established	GM diversity was associated with the response to TTM < diversity < response	Regulation of cholesterol, BA, and lipid metabolism.
	**ROSUVASTATIN**				
Kummen *et al.* [156]	20 mg/d (6 months)	Humans	Minimal effects on GM	< Response to TTM in individuals with > TMAO concentrations	Regulation of TMAO metabolism
Nolan *et al.* [157]	9.3 mg/kg/d (28 days)	C57Bl/6 female mice receiving a Western diet	↑ *Coprococcus* and *Rikenella genera* and L*achnospiraceae* family ↓ *Roseburia* and *Erysipelotrichaceae* genera, and RF9 family	Not established	Regulation of cholesterol, lipids, and BA metabolism
Liu *et al.* [158]	10 mg/d (4-8 wk)	Patients with hyperlipidemia	Not established	> Firmicutes and BPB families of Clostridiaceae Ruminococcaceae and Lachnospiraceae abundance and < Bacteroidetes was associated with the optimal ROSU effect	Regulation of cholesterol, lipids, BA metabolism, and endotoxemia
	**SIMVASTATIN**				
Zhang *et al.* [152]	20 mg/Kg/d (8 wk)	Mice with hyperlipidemia	↓ Gut dysbiosis	Not established	Regulation of BA and cholesterol metabolism ↑ SCFA
Kaddurah-Daouk *et al.* [150]	4 0mg/day (6 wk)	Patients with hypercholesterolemia	Not established	Pre-treatment BA levels predicted TTM response Individuals with > levels of coprostanoligenes bacteria pre-treatment had a better response to SIM	Competition between SIM and BA for the SLCO1B1 hepatic transporter
	**ATORVASTATIN OR ROSUVASTATIN**				
Kim *et al.* [153]	ATO 10 mg/kg/d ROSU 3mg/kg/d (16 sem)	Male mice receiving a fat-rich diet	↑Bacteroides, Butyricimonas and Mucispirillum.	Not established	Regulation of the inflammatory response ↑ SCFA
	**ROSUVASTATIN, SIMVASTATIN, FLUVASTATIN OR ATORVASTATIN**				
Zhao *et al.* [159]	ROSU 30; SIM 40, FLU 240 y ATO 64 μg/m	*In vitro*	↓ Bacteroidetes and ↑ of *Escherichia/ Shigella, Sutturella,* and Ruminococcaceae abundant in the FLU fermentation samples	Degradation and/or modification by all statins	↑ SCFA

**Abbreviations:** BA: bile acids; SCFA: short-chain fatty acids; ATO: atorvastatin; CVD: cardiovascular disease; FLU: fluvastatin; GM: gut microbiome; ROSU: rosuvastatin; SIM: simvastatin; TMAO: trimethylamine N-oxide; TTM: treatment.

## LIST OF ABBREVIATIONS

GM: Gut Microbiome
CVD: Cardiovascular Disease
TRIF: TIR-domain-containing Adapter-inducing Interferon-β
TRAM: TRIF-related Adaptor Molecule
INF: I Interferon
